# Unexpected impairment of TNF-α-induced maturation of human dendritic cells in vitro by IL-4

**DOI:** 10.1186/s12967-016-0848-2

**Published:** 2016-04-14

**Authors:** Valérie Chabot, Laurence Martin, Daniel Meley, Luc Sensebé, Christophe Baron, Yvon Lebranchu, Frédéric Dehaut, Florence Velge-Roussel

**Affiliations:** Service Recherche du laboratoire d’Histocompatibilité et d’Immunogénétique, Etablissement Français du Sang Centre Atlantique, Tours, France; UFR de Médecine, UPRES EA 4245 “Cellules Dendritiques, Immunomodulation et Greffes”, Université François-Rabelais de Tours, 10 Boulevard Tonnellé, 37032 Tours, France; STROMALab UMR 5273 UPS/CNRS/EFS/Inserm U1031, Toulouse, France; Service de Néphrologie et d’Immunologie Clinique, CHRU de Tours, 2bis Boulevard Tonnellé, 37000 Tours, France

**Keywords:** Dendritic cell, Maturation, Migration, Immune function, Immunotherapy

## Abstract

**Background:**

An efficient strategy for programing dendritic cells (DCs) for cancer immunotherapy is the optimization of their maturation so that they can efficiently stimulate cancer-specific T cell responses. Interleukin (IL)-4 has appeared as an essential cytokine, widely used in vitro with granulocyte macrophage-colony stimulating factor (GM-CSF) to differentiate monocytes into immature DCs (iDC) and to prevent macrophage formation. Conflicting data have been published regarding the effect of IL-4 on functional DC maturation. To further understand IL-4’s effects on DC maturation and function in vitro, we choose the most commonly used maturation factor tumor necrosis factor (TNF)-α.

**Methods:**

Human monocyte-derived iDC were treated for 48 h with GM-CSF and TNF-α in the presence (IL-4^+^-DC) or absence (IL-4^−^-DC) of IL-4 and functions of both DC populations were compared.

**Results:**

On mixed lymphocyte reaction assay, IL-4^+^-DC were less potent than IL-4^−^-DC at inducing the proliferation of allogeneic CD4^+^ T cells and the proportion of activated T cells expressing CD69 and/or CD25 was smaller. Interleukin-4 reduced the cell-surface expression of TNF-α-induced DC maturation markers CD83, CD86, HLA-DR and CD25 and generated a heterogeneous population of DCs. IL-4^+^-DC secreted less IL-12 and more IL-10 than IL-4^−^-DC following activation by soluble CD40L, and IL-4^+^-DC-activated T cells secreted lesser amounts of T helper (Th) 1 cytokines (IL-2 and interferon-γ). Importantly, IL-4 impaired the in vitro migratory capacity of DCs in response to CCL21 and CCL19 chemokines. This effect was related to reduced expression of CCR7 at both mRNA and protein levels.

**Conclusion:**

Interleukin-4 used with GM-CSF and TNF-α during the maturation of DCs in vitro impaired DC functions and disturbed the maturation effect of TNF-α. Finally, our study reinforces the view that the quality of the DC maturation stimulus, which regulates DC migration and cytokine production, may be a decisive feature of the immunogenicity of DCs.

## Background

Dendritic cells (DCs) are the most potent antigen-presenting cells (APC) and play a pivotal role in the initiation of the primary immune response [1]. They are generated in vitro in large quantities from peripheral blood monocytes and are commonly used in active cancer immunotherapy. Results obtained over the last 15 years have highlighted the poor clinical efficacy of DC-based vaccine, related essentially to an ineffective migration of injected DCs to the peripheral lymphoid organs and insufficient T-cell help secondary to inadequate antigen presentation by major histocompatibility complex (MHC) class II [2]. Today, a large number of several strategies have developed; some using DC targeting combined with chemotherapy or agonists of TLRs, others interested by DC subsets [3]. Whatever these strategies, monocyte-derived DCs appeared always as one relevant actor in cancer immunotherapy [4].

The manner by which DCs are matured in vitro is clearly an important variable that governs their subsequent functionality. Upon maturation, DCs up-regulate the expression of molecules such as CD80 and CD86 for co-stimulation, as well as MHC, and produce cytokines that are instructive signals mirroring the micro-environment in which they were activated [5]. These different stimuli contribute to differential levels of T cell activation and T helper (Th) polarization of the immune response [6]. For the design of DC-based vaccines for immunotherapy against tumors, the challenge is to find the most potent source of DCs and the appropriate cytokine milieu for maturation to induce Th1-cell differentiation [7, 8]. The most widely used protocols for maturation of clinical grade monocyte-derived DC include the use of granulocyte macrophage-colony stimulating factor (GM-CSF) and interleukin (IL)-4 in combination with tumor necrosis factor (TNF)-α alone or with IL-1β, IL-6 and prostaglandin (PG) E2, also known as “the maturation cocktail” [9, 10]. This cocktail was challenge because of low production of IL-12p70 and the induction of Th2-type immune responses. TNF-α used alone is a well-known factor able to induce high expression levels of MHC class II and co-stimulatory molecules on DCs but is known as a weak stimulator of IL-12 production, CCR7 expression and DC migration [11]. However at least in mice, dendritic cells matured with TNF-α can be further activated in vitro and after subcutaneous injection in vivo a process that converts their tolerogenicity into immunogenicity [12].

In many cell types, it is well known that IL-4 has anti-TNF-α and anti-inflammatory effects [13]. Interleukin-4 is necessary for the differentiation of monocytes into immature DCs (iDC) in vitro. It has been demonstrated that monocytes cultivated with GM-CSF and TNF-α alone from the beginning of the culture, were converted to CD14 positive/CD1a low-adherent cells with a lower capacity to stimulate T cells [14]. Moreover, the use of IL-4 with GM-CSF during the differentiation step of monocytes into iDC has been shown to overcome the problem of donor diversity, which results from the variability in GM-CSF receptor alpha expression, and allowed a more homogeneous population of iDC to be generated [15].

Conflicting data have been published regarding the effect of IL-4 on functional DC maturation. IL-12p70 production by DCs is increased by IL-4 in lipopolysaccharide (LPS)- and CD40L-matured DC [16]. Other studies have reported that the use of IL-4 to generate DC for therapeutic use could be inappropriate if the objective is to induce long-term Th1 responses [17]. Furthermore, a few studies have suggested that continuous, high concentrations of IL-4 during DC maturation with LPS [18] or polyinosinic:polycytidylic acid (poly I:C) [19], may generate disabled DCs through suppression of the mobilization of endogenous PGE2. Moreover, migration capacity of DCs could also be impaired in the presence of IL-4 as observed on human Langerhans cells by down regulation of TNF-R II [20].

To further understand IL-4’s effects on DC maturation and function in vitro, we choose the most commonly used maturation factor tumor necrosis factor (TNF-α) and we hypothesized that the combined use of IL-4 and TNF-α during the maturation step of DC might be responsible for their phenotypic and functional deficiency. A better understanding of the role of cytokines in the differentiation and maturation of DC could lead to promising new developments in the design of more effective and rational DC-vaccine strategies.

## Methods

### Generation of IL-4^−^ and IL-4^+^ DC

Human iDC were generated in vitro as previously described [21]. Peripheral blood mononuclear cells (PBMC) were obtained via cytapheresis, from the blood of healthy volunteers who had given their written informed consent and the University ethic committee approved the procedure. Adherent monocytes were cultured in RPMI-1640 medium supplemented with 10 % (v/v) heat-inactivated fetal calf serum (FCS), 2 mM l-glutamine, 50 U/ml penicillin and 50 µg/mL streptomycin (Invitrogen, Cergy-Pontoise, France) supplemented with 1000 U/mL recombinant human (rh) GM-CSF (AbCys S.A., Paris, France) and 25 ng/mL rh IL-4 (R&D Systems Europe, Abingdon, UK) for 5 days. Fresh medium containing rhGM-CSF and rhIL-4 was added on Day 3 (0.5 volume). Immature DC were extensively washed with RPMI and then stimulated by the addition of 20 ng/mL TNF-α (R&D Systems Europe) for 48 h, in the absence (IL-4^−^-DC) or presence (IL-4^+^-DC) of 25 ng/mL IL-4. For the IL-4 and IL-13 dose-dependent experiments, iDC were activated with GM-CSF, TNF-α and 0, 0.5, 1, 5 or 25 ng/mL IL-4 or IL-13 (R&D Systems Europe) for 48 h. For time-dependent experiments they were activated for 0, 8, 24 or 48 h in the absence or presence of 25 ng/mL IL-4. For the IL-4 effect on TNF-α, LPS or sCD40L-induced CCR7 cell-surface expression, iDC were treated with GM-CSF and 20 ng/mL TNF-α, 50 ng/mL LPS (Sigma-Aldrich, Saint Quentin Fallavier, France) or 250 ng/ml sCD40L pre-incubated with its enhancer (Alexis Biochemicals, San Diego, CA, USA) in the presence or absence of 25 ng/mL IL-4. The cells were then harvested. Viability, measured by a Trypan blue exclusion test, was found to be greater than 95 %. The purity of the DC preparations, determined by flow cytometry analysis of CD1a and DC-SIGN expression on total cultured cells, was also greater than 95 %.

### FACS analysis

Interleukin-4^−^ and IL-4^+^ DC, obtained after 7-day culture, were labeled with monoclonal antibodies (mAbs) for human DC-SIGN (clone AZND1), CD1a (clone HI149), CD83 (clone HB15a), CD86 (clone HA5.2B7), HLA-DR (clone B8.12.2; all from Beckman Coulter, Roissy, France), CD25 (clone M-A251; BD Biosciences, Le pont de Claix, France), CCR7 (clone 150503; R&D Systems Europe), conjugated with fluorescein isothiocyanate (FITC), phycoerythrin (PE) or allophycocyanin (APC). Cells were also stained with the corresponding FITC-, PE- or APC-conjugated isotype-matched control mAbs.

For CCR7 intracellular staining (rat PE-anti-CCR7 mAb, clone 3D12; BD Biosciences), cells were pre-incubated for 30 min at 37 °C with purified anti-CCR7 mAb (clone 150503) to neutralize cell-surface expression of CCR7. The cells were then fixed and permeabilized using IntraPrep Permeabilization Reagent (Beckman Coulter) according to the manufacturer’s instructions.

Human CD4^+^ T cells, co-cultured with IL-4^−^ or IL-4^+^-DC for 5 days, were stained with FITC-anti-CD3 (clone UCHT1), PE-anti-CD69 (clone TP1.55.3; both from Beckman Coulter) and APC-anti-CD25 mAbs. Intracellular staining with APC-anti-interferon (IFN)-γ (clone B27; BD Biosciences) mAb was performed at days 5 and 6 of the co-culture, after a 4-h incubation of the cells with Golgi Stop (BD Biosciences) and IntraPrep Permeabilization Reagent (see above). At least 5000–10,000 cells were obtained, measured using a 488-nm laser flow cytometer (FACSCalibur, BD Biosciences). Data were analyzed using CELLQuest^®^ software (BD Biosciences). The results are expressed as the percentage of labeled cells or as the ratio of mean fluorescence intensity (MFI) of specific labeling to background staining.

### Allogeneic mixed lymphocyte reaction (MLR)

Allogeneic human CD4^+^ T cells were purified using a CD4-positive isolation kit (Dynal France SA, Compiègne, France) as described [22]. CD4^+^ T cells (1 × 10^5^/well) were co-cultured in triplicate with an increasing number (1 × 10^4^, 3 × 10^4^, 1 × 10^5^/well) of IL-4^−^ or IL-4^+^ DC in 96-well plates, over a five-day period. Dendritic cells alone (1 × 10^5^/well) were used as a control. T-cell proliferation was evaluated by the addition of 0.5 µCi/well of ^3^H thymidine (Amersham, France) 18 h before the end of the co-culture. Radioactivity was quantified on a β counter (Tri-Carb 2550, Packard, Rungis, France).

### Cytokine detection by enzyme-linked immunosorbent assay (ELISA)

For the determination of IL-12p70 and IL-10 release, IL-4^−^ and IL-4^+^-DC were stimulated for 24 h with 250 ng/mL recombinant sCD40L (Alexis Biochemicals). Supernatants were collected and stored at −80 °C. The IL-12p70 and IL-10 concentrations were determined by ELISA (eBioscience, San Diego CA, USA), according to the manufacturer’s instructions. The cytokines in the supernatants were measured in the MLR assay using a specific ELISA for IL-2, IFN-γ, and IL-5 (eBioscience), according to the manufacturer’s instructions.

### RT-Rt-PCR

The total mRNA was isolated from 5 × 10^5^ IL-4^−^ or IL-4^+^ DC by use of the Dynabeads mRNA Direct kit (Dynal France SA), according to the manufacturer’s instructions. This total mRNA was then reverse-transcribed for 1 h at 45 °C in 1X incubation buffer containing 250 µM deoxynucleotide triphosphate, 5 µM oligo (dT)_20_, 12 units of RNase inhibitor and 10 units of AMV Reverse Transcriptase (Invitrogen, France).

Standard PCR was then performed with cDNA obtained from 5 × 10^4^ cells in a total reaction volume of 50 µL, containing 10 mM Tris–Hcl, pH 9.0, 50 mM KCl, 0.01 % (w/v) gelatin, 1.5 mM MgCl_2_, 0.1 % Triton X-100, 50 µM deoxynucleoside triphosphate, 1 µM of forward- and reverse-synthesized oligonucleotide primers (Invitrogen), and 1 unit Super T*aq*® DNA polymerase (A.T.G.C. Biotechnologie, Noisy-le-Grand, France). The primers used for the PCR of CCR7 and for β-actin (Sigma-Proligo), acting as a control, were as follows: forward, 5′-TCCTTCTCATCAGCAAGCTGTC-3′, and reverse, 5′-CTTCAAGGACCTGGGCTGCCTC-3′; and forward, 5′-AGCGGGAAATCGTGCGTG-3′, and reverse, 5′-GGCACCACCATGTACCCTG-3′, respectively. The amplification reaction included denaturation at 94 °C for 5 min followed by 30 cycles at 94 °C for 30 s, 53 °C for 30 s and 72 °C for 1 min, and a final extension step at 72 °C for 7 min. The MyCycler Thermal Cycler system (Biorad, Marnes la Coquette, France) was used for this reaction. The amplified fragments were size-separated on 1.6 % agarose gel and visualized by ethidium bromide staining. The band intensities of PCR products were measured by use of Image J software (US National Institutes of Health).

### Chemotaxis assay

The chemotaxis of IL-4^−^ and IL-4^+^ DC to rhCCL21 and rhCCL19 (R&D Systems) was assayed in 24-well cell culture plates with bare 8.0-µm-pore polycarbonate cell-culture inserts (Corning BV, Schiphol-Rijk, The Netherlands). Dendritic cells were washed extensively in RPMI-1640 medium to remove FCS and re-suspended in RPMI-1640 containing 1 % (v/v) human serum albumin (HSA, LFB, France) at a concentration of 5 × 10^5^ cells/100 µL. For optimal induction of DC migration, 600 µL of RPMI 1640-1 % (v/v) HSA alone, or with rhCCL21 or rhCCL19 at 500 or 1000 ng/mL, was added to the wells. Each condition was set up in duplicate. A100 µL cell suspension (5 × 10^5^ DC) in RPMI-1 % HSA was then added to the inserts and the plates were incubated for 5 h at 37 °C, in 5 % CO_2_. The cells remaining in the inserts were subsequently removed; aspiration and wiping with cotton harvested swabs, and migrated cells by washing the base of the filter insert and the wells with 600 µL RPMI-1640. Migrated cells were counted using a Malassez chamber. The number of cells that spontaneously migrated towards the medium alone (without chemokines) was deduced from specific migration toward medium with chemokines. Results are expressed as the mean number of migrating cells ± SEM.

### Statistical analysis

The Mann–Whitney or Wilcoxon signed-rank test assessed the significance of differences between means using Statview software (SAS Institute, Berkeley, CA). A *P* < 0.05 was considered statistically significant.

## Results

### IL-4^+^-DC show reduced capacity for stimulation of allogeneic CD4^+^ T-cells

**Fig. 1:**
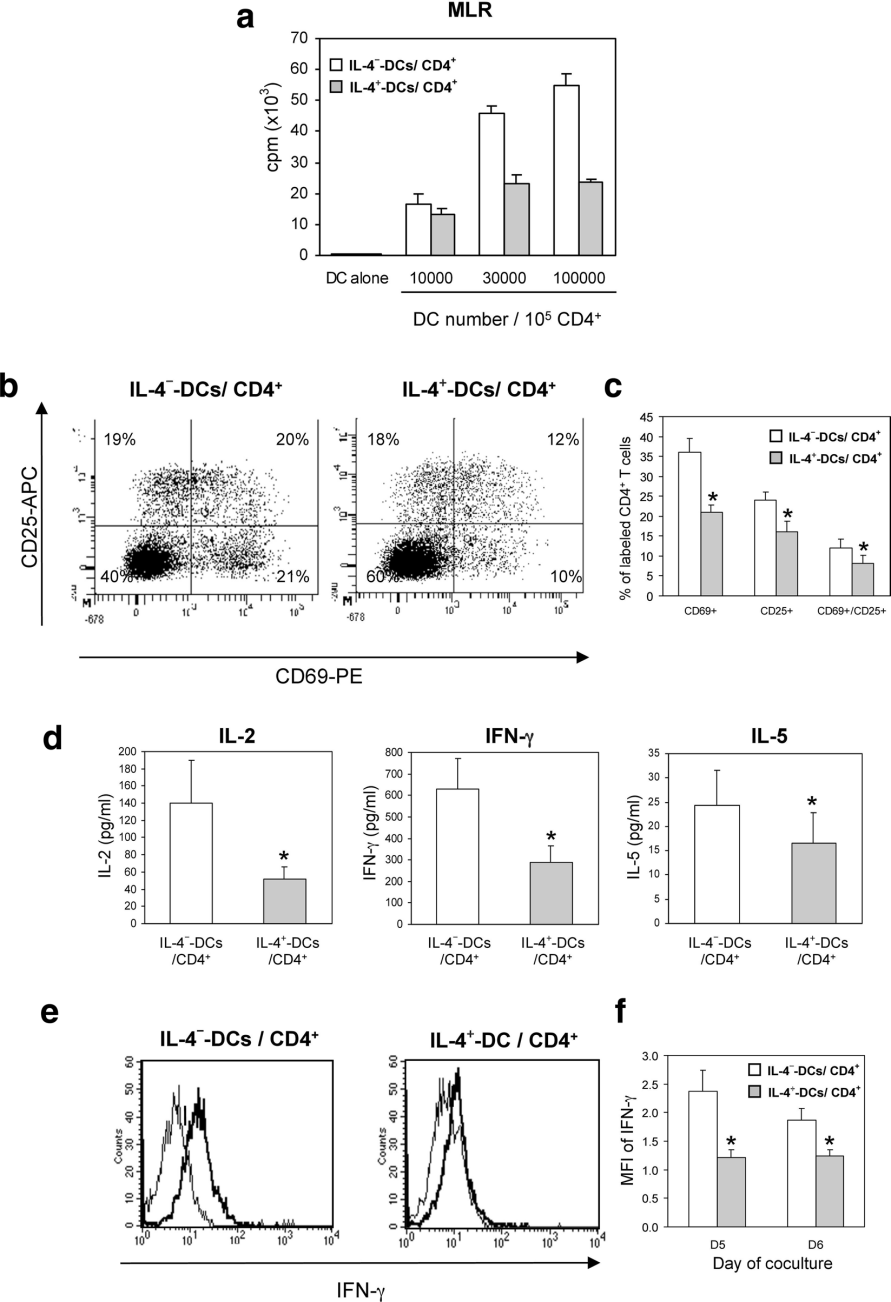
IL-4^+^-DC have reduced capacity to induce proliferation and activation of allogeneic CD4^+^ T cells. **a** In the MLR assay, allogeneic CD4^+^ T cells (1 × 10^5^/100 µL) were co-cultured with increasing numbers of IL-4^−^ or IL-4^+^-DC (1 × 10^4^, 3 × 10^4^ or 1 × 10^5^/10 µL) for 5 days. Dendritic cells alone (1 × 10^5^/100 µL) were used as a control. Proliferation was measured by the addition of ^3^H thymidine 18 h before the end of the co-culture and radioactivity was quantified on a β counter. The results are representative of three independent experiments. **b** Allogeneic CD4^+^ T cells (1 × 10^5^/100 µL), co-cultured with IL-4^−^ or IL-4^+^-DC (3 × 10^4^/100 µL) for 5 days, were removed, stained with specific mAb for CD69 and CD25, or their isotype controls, and analyzed by flow cytometry. Labeled cells were gated on CD3^+^/DC-SIGN^−^ cells. **c** The results are expressed as the mean ± SEM  % of CD4^+^ T cells expressing the indicated markers of six independent experiments. *P < 0.05, compared to IL-4^−^-DC/CD4^+^. **d** IL-4^−^ and IL-4^+^-DC (3 × 10^4^/100 µL) were co-cultured with allogeneic CD4^+^ T cells (1 × 10^5^/100 µL) for 5 days and the supernatants were analyzed for IL-2, IFN-γ, and IL-5, using ELISA. The results are the mean ± SEM of six to eight separate experiments. *P < 0.05, compared to IL-4^−^-DC/CD4^+^. **e** Flow cytometry analysis of intracellular IFN-γ content. CD4^+^ T cells were fixed, permeabilized and stained with APC-anti-IFN-γ mAb (bold lines) or its isotype control (*thin lines*). The histograms are representative of five experiments. **f** MFI of IFN-γ labeling calculated from the total fraction of CD4^+^ T cells at days 5 and 6 of co-culture. The results are the mean ± SEM of the MFI of labeled cells from four to five separate experiments. *P < 0.05, compared to IL-4^−^-DC/CD4^+^

To evaluate the effect of IL-4 on DC function, we used IL-4^−^- or IL-4^+^-DC as stimulating cells in co-culture with allogeneic CD4^+^ T cells for MLR assay. Co-culture of IL-4^−^-DC with CD4^+^ T cells induced allogeneic T-cell proliferation that strongly increased with DC number (max 1:1 DC/T-cell ratio). Conversely, this response was impaired when high concentrations of IL-4^+^-DC were used (Fig. 1a). Dendritic cells cultured alone did not proliferate, which verified that the assay detected only the allogeneic T-cell responses (Fig. 1a). On flow cytometry analysis, IL-4^+^-DC induced a lower percentage of activated CD4^+^ T cells expressing CD69 and/or CD25 than did IL-4^−^-DC (40 versus 60 %; Fig. 1b, c). Analysis of each CD4^+^ T-cell sub-population showed a strong effect of IL-4^+^-DC on CD69 expression, causing it to be reduced almost by half (Fig. 1b, c).

### IL-4 decreases the Th1 cell-inducing potential of TNF-α-matured DCs

The orientation of the immune response induced by IL-4^−^ or IL-4^+^-DC was evaluated by the production of lymphocytes Th1 (IFN-γ, and IL-2), Th2 (IL-5) and Treg (IL-10)-associated cytokines into the co-culture supernatants from allogeneic MLR. Interleukin-4^−^-DC induced a strong secretion of IFN-γ and IL-2 in the co-culture supernatants that was reduced by two- or three-fold when IL-4^+^-DC were used (Fig. 1d). The secretion of IL-5 was weakly detected in both IL-4 conditions, values being less than 30 pg/mL, and was significantly weaker on IL-4^+^-DC/CD4^+^ co-culture (Fig. 1d). Since IFN-γ may be secreted by DCs themselves, intracellular staining for this cytokine was performed on CD4^+^ T cells and analyzed by flow cytometry (labeled cells were gated on CD3^+^/DC-SIGN^−^ cells). Interleukin-4^−^-DC induced approximately 50 % of T cells positive for intracellular IFN-γ (Fig. 1e). The percentage of IFN-γ-positive CD4^+^ T cells and MFI were both reduced by half when IL-4^+^-DC were used in the co-culture (Fig. 1e, f). This effect was significant at days 5 and 6 of the co-culture (P < 0.05; Fig. 1f). However, less than 1 % of CD4^+^ T cells positive for intracellular IL-4 were detected by flow cytometry, even after stimulation with PMA and ionomycin at the end of the co-culture with both types of IL-4-DC (data not shown). Interleukin-10 was weakly detected in both conditions, with no significant difference in secretion (data not shown).

### IL-4 reduces the expression of maturation markers and IL-12 secretion of TNF-α-treated DCs

**Fig. 2:**
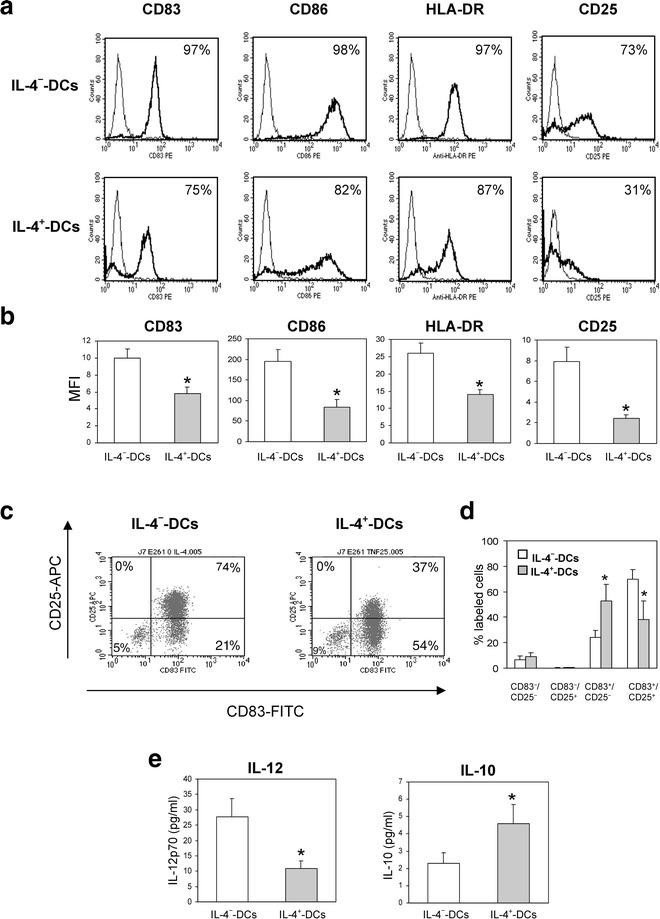
IL-4 impairs TNF-α-induced cell-surface expression of DC maturation markers. Dendritic cells were derived from human monocytes cultured for 5 days with GM-CSF (1000 IU/mL) and IL-4 (25 ng/mL). Maturation was induced after an additional 48-h culture with GM-CSF and 20 ng/mL TNF-α, in the absence (IL-4^−^) or presence (IL-4^+^) of 25 ng/mL IL-4. **a** Cells were stained with specific mAbs directed against CD83, CD86, HLA-DR or CD25 (*bold lines*) or their respective isotype controls (*thin lines*) and analyzed by flow cytometry. The figure in each histogram indicates the percentage of positive cells. The results are representative of 10 independent experiments. **b** Mean fluorescence intensity (MFI) for CD83, CD86, HLA-DR and CD25 expressed as the ratio of fluorescence intensity of specific labeling to background staining. The results are the mean ± SEM of MFI of ten separate experiments. *P < 0.05, compared to IL-4^−^-DC. **c** Cells were double-stained with FITC-anti-CD83 and APC-anti-CD25 antibodies and analyzed by flow cytometry. **d** The results are expressed as the mean ± SEM  % of IL-4^−^ and IL-4^+^-DC expressing the indicated markers in five separate experiments. *P < 0.05, compared to IL-4^−^-DC. **e** IL-4^−^ and IL-4^+^-DC were stimulated during an additional 24-h culture with 250 ng/mL recombinant soluble CD40L and underwent ELISA for IL-12p70 and IL-10 release. The results are the mean ± SEM of six to eight independent experiments. *P < 0.05, compared to IL-4^−^-DC

To understand the decreased capacity of DC with regard to allogeneic T cell stimulation, the cells were cultured for 48 h with GM-CSF and TNF-α in the absence or presence of IL-4. Flow cytometry analysis revealed that 100 % of DC populations were CD11c + (data not shown). IL-4^−^-DC expressed the maturation markers CD83, CD86 and HLA-DR, with almost 100 % of cells being positive (Fig. 2a). A lower percentage of IL-4^+^ than IL-4^−^ DC were positive for CD83, CD86, HLA-DR and CD25 (Fig. 2a), and the MFI for all these markers was also lower (P < 0.05; Fig. 2b). The latter effect was dose-dependent (data not shown) and varied depending on the donor.

The majority of cells were CD83^+^/CD25^+^ in IL-4^−^ DC as shown by analyses of the double staining CD83/CD25 population (Fig. 2c). Treatment with IL-4 induced a significant decrease in CD83^+^/CD25^+^ DC whereas it increased CD83^+^/CD25^−^ DC (P < 0.005, Fig. 2d).

Since the secretion of IL-12p70 could not be detected after treatment with TNF-α alone (data not shown), Interleukin-4^−^ and IL-4^+^-DC were stimulated for 24 h with recombinant sCD40L, thus mimicking a T cell encounter. ELISA revealed that IL-4^+^-DC secreted less IL-12p70 and more IL-10 than did IL-4^−^-DC (P < 0.05) (Fig. 2e). Taking these results together, Interleukin-4 appeared to have an inhibitory effect on the maturation marker expression and Th1 orientation profile capacity of DC.

### IL-4 decreases TNF-α matured DC migration toward CCL21 and CCL19

**Fig. 3:**
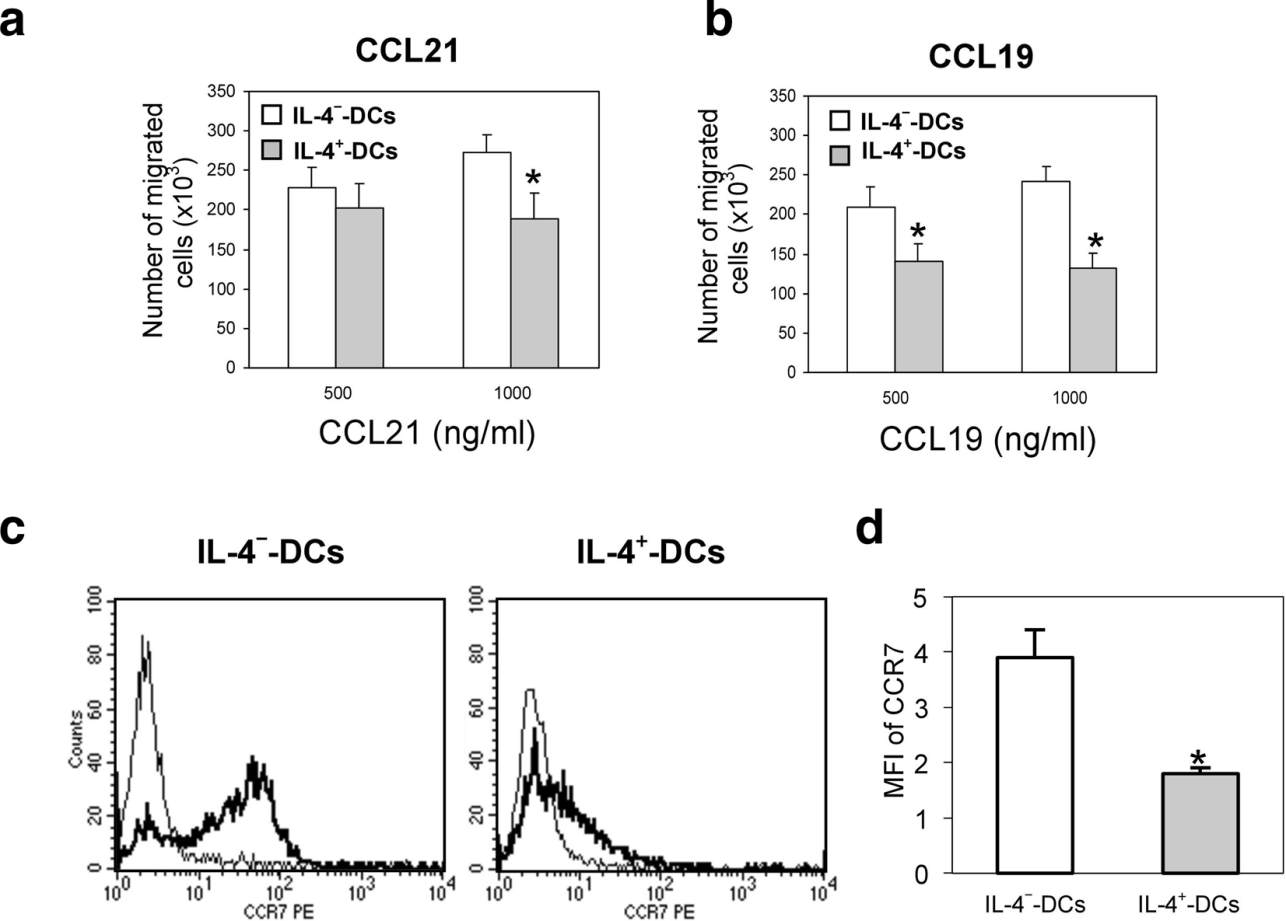
IL-4 impairs the migration of TNF-α-matured DC towards CCL21, CCL19 and CCR7 expression. **a**, **b** The chemotactic migration of IL-4^−^ and IL-4^+^-DC in response to rhCCL21 and rhCCL19 (500 or 1000 ng/mL) was assayed in vitro. Serum-free medium, either alone or with CCL21 or CCL19, was added to the wells, followed by 5 × 10^5^ DC. For each experiment, non-specific migration in medium alone was deducted from specific migration in response to each chemokine. The data represent the mean ± SEM of migrated DC from five separate experiments. *P < 0.05, compared to IL-4^−^-DC. **c** IL-4^−^ and IL-4^+^-DC were stained with PE-anti-CCR7 antibody (*bold lines*) or its isotype control (*thin lines*) and analyzed by flow cytometry. The results are representative of 11 independent experiments. **d** Mean fluorescence intensity (MFI) of surface CCR7 content expressed as the ratio of fluorescence intensity of specific labeling to background staining. The results are the mean ± SEM of the MFI of six independent experiments

As migratory capacity is an important competence of DCs in immunotherapy, we investigated the effect of IL-4 on the chemotactic migration of DCs toward the CCR7 ligands CCL21 and CCL19. In the absence of chemokines (medium alone), IL-4^+^-DC showed an unspecific migration greater than that of IL-4^−^-DC (data not shown). Interleukin-4^−^-DC exhibited a strong chemotactic response to both CCL21 and CCL19, which was optimal at a concentration of 1000 ng/mL (Fig. 3a, b). Treatment with IL-4 significantly reduced the migration of DC to both chemokines, the effect being maximal at 1000 ng/mL, with up to 45 % inhibition of CCL19-induced chemotaxis (Fig. 3b).

### IL-4 decreases the CCR7 up-regulation in TNF-α-maturated DC

**Fig. 4:**
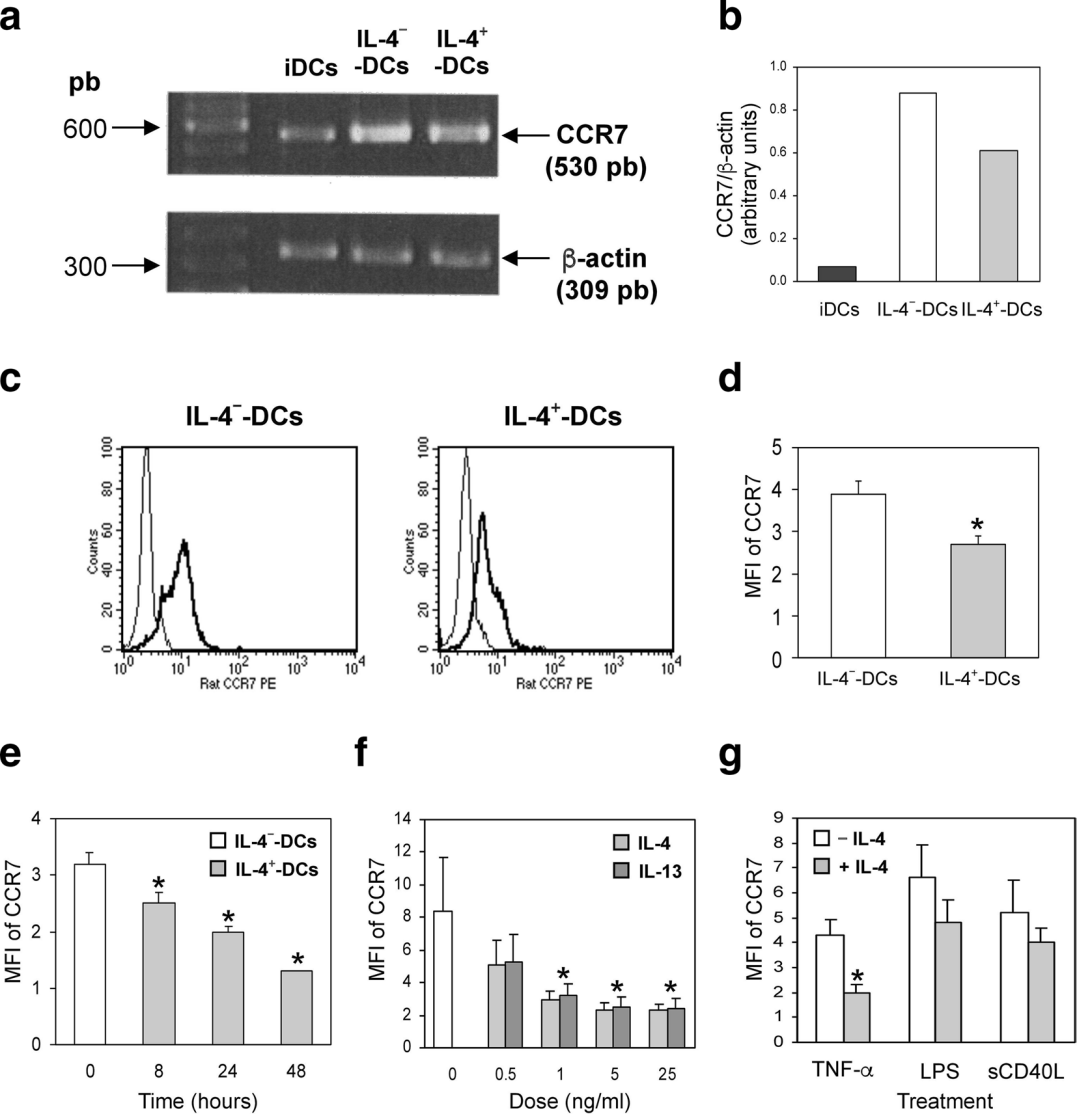
IL-4 reduces CCR7 mRNA synthesis, intracellular and surface protein content in TNF-α-matured DC. **a** RT-PCR analysis of CCR7 and β-actin mRNA expression in 5 × 10^4^ iDC, IL-4^−^ and IL-4^+^-DC. **b** Quantification of RT-PCR analysis in (**a**). Data are expressed as the ratio of expression of CCR7 to β-actin. The results are representative of four independent experiments. **c** Interleukin-4^−^ and IL-4^+^-DC were fixed, permeabilized and stained with rat PE-anti-CCR7 antibody (bold lines) or its isotype control (thin *lines*) and analyzed by flow cytometry. The results are representative of six independent experiments. **d** Mean fluorescence intensity (MFI) of CCR7 intracellular content expressed as the ratio of fluorescence intensity of specific labeling to background staining. The results are the mean ± SEM of the MFI from six independent experiments. **e** Immature DC were cultured for 0, 8, 24 or 48 h with IL-4 (25 ng/mL) during maturation with GM-CSF and TNF-α. **f** Immature DC were cultured for 48 h, either in the absence of (0) or with increasing concentrations (0.5, 1, 5, 25 ng/mL) of IL-4 or IL-13, during maturation with GM-CSF and TNF-α. **g** Immature DC were activated with GM-CSF and TNF-α (20 ng/mL), LPS (50 ng/mL) or sCD40L (250 ng/mL) in the absence or presence of 25 ng IL-4. **e**–**g** Cells were stained with PE-anti-CCR7 antibody and analyzed by flow cytometry. The results are the mean ± SEM of the MFI from four to five separate experiments. *P < 0.05, compared to DC activated in the absence of IL-4

CCR7 is a crucial receptor involved in DC function in vivo because it governs cell migration to the draining lymph nodes. Flow cytometry analysis revealed that IL-4^−^-DC were strongly positive for CCR7 cell-surface expression (Fig. 3c, d). The addition of IL-4 inhibited the TNF-α-induced up-regulation of CCR7 expression by two- or threefold (Fig. 3d). RT-PCR analysis revealed that iDC synthesized very low levels of CCR7 mRNA, however the expression was strongly increased in IL-4^−^-DC after 48-hour treatment with TNF-α and GM-CSF (Fig. 4a, b). In the presence of IL-4, TNF-α-induced up-regulation of CCR7 mRNA synthesis was impaired, which resulted in lower CCR7 intracellular protein content in IL-4^+_^ than in IL-4^−^-DC (P < 0.05; Fig. 4c, d). Time dependent experiments with 25 ng/mL of IL-4 revealed an inhibitory effect after only 8 h of treatment, which subsequently increased along the incubation time (Fig. 4e). This effect was dose-dependent and was observed not only with an IL-4 dose of 25 ng/mL but also with as little as 1 ng/mL (Fig. 4f). Replacing IL-4 with IL-13 had the same dose-dependent inhibitory effect on CCR7 cell-surface expression (Fig. 4f). To investigate whether the inhibitory effect of IL-4 on CCR7 expression was limited to TNF-α-treated DCs, other stimuli were included in this study. Treatment with LPS or sCD40L for 48 h did not significantly reduce the IL-4 inhibition of CCR7 expression (Fig. 4g).

### IL-4 induces an immature profile of DCs associated with lower CCR7 expression

**Fig. 5:**
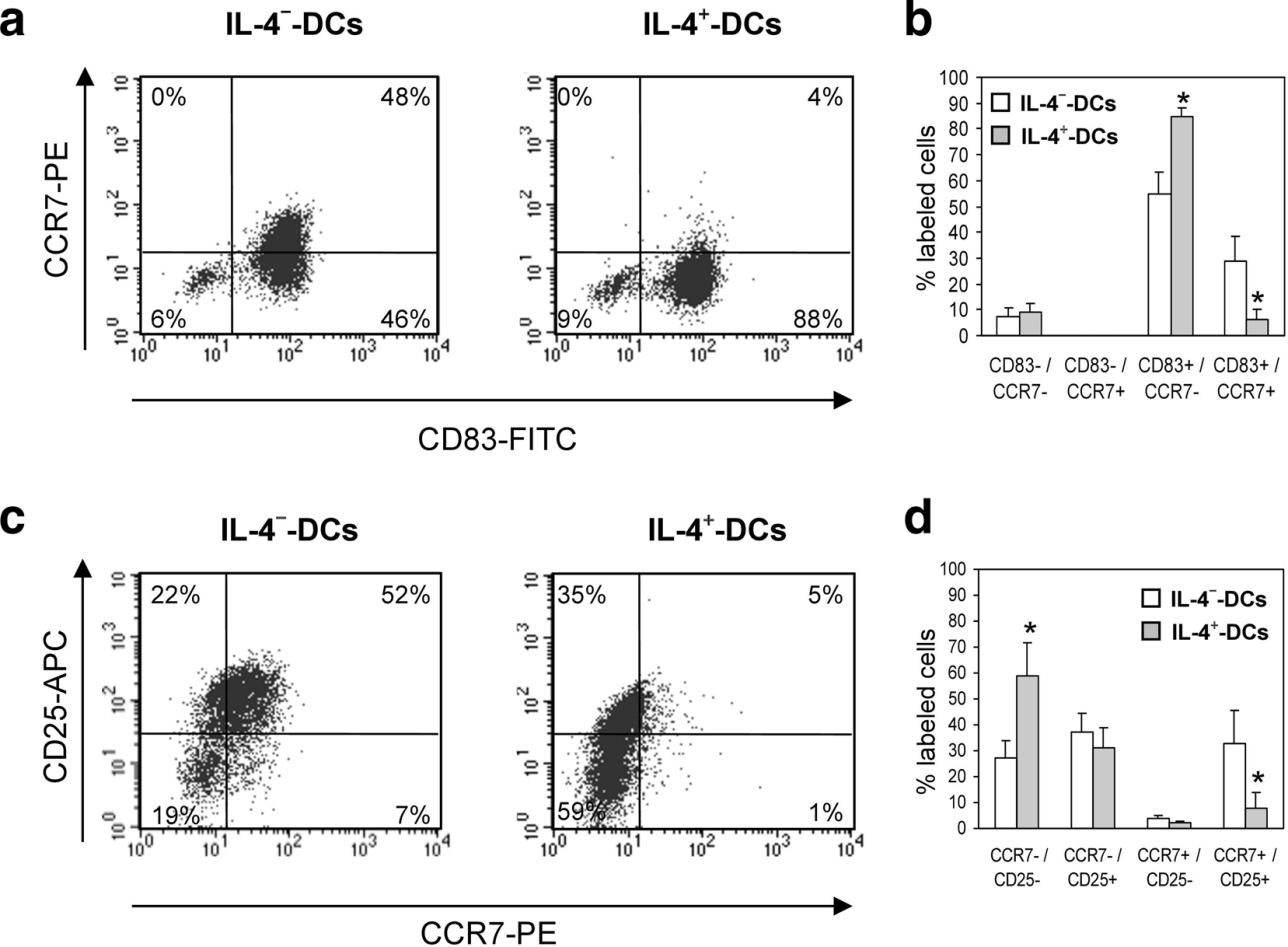
IL-4 reduces the percentage of DC that are double positive for CCR7 and CD83 or CD25 induced by TNF-α. IL-4^−^ and IL-4^+^-DC were double-stained with PE-anti-CCR7 and FITC-anti-CD83 (**a**, **b**) or APC-anti-CD25 mAbs (**c**, **d**) and analyzed by flow cytometry. The results of double labeling for CCR7 and CD83 (**b**) or CCR7 and CD25 (**d**) are expressed as the mean ± SEM  % of IL-4^−^ and IL-4^+^-DC expressing the indicated markers in five separate experiments. *P < 0.05, compared to IL-4^−^-DC

To evaluate precisely which DC population was expressing a lower amount of CCR7, staining for CD83, CD25 and CCR7 was performed on IL-4^−^ and IL-4^+^-DC. Double staining of IL-4^−^- and IL-4^+^-DC for CD83 and CCR7 revealed that all CCR7^+^ cells were CD83^+^ in the five tested donors, since no CD83^−^/CCR7^+^ cells were detected (Fig. 5a, b). Interleukin-4 treatment decreased the percentage of CD83^+^/CCR7^+^ cells and increased the percentage of CD83^+^/CCR7^−^ cells (P < 0.05; Fig. 5b). Thus, the extent of CCR7 inhibition strongly exceeded that of CD83 inhibition. Double staining for CCR7 and CD25 revealed that almost 50 % of IL-4^−^-DC were CCR7^+^/CD25^+^ (Fig. 5c, d). Treatment with IL-4 strongly decreased this cell population and significantly enhanced the percentage of CCR7^−^/CD25^−^ cells (P < 0.05; Fig. 5d). Overall, IL-4 is in favor of a CD83^+^/CD25^−^/CCR7^−^ DC profile.

## Discussion

Ex vivo-generated DCs have been used as therapeutic vaccines in patients with cancer for over a decade [6]. Increasing knowledge on DC subsets and maturation is leading to a better definition of the essential parameters for DC therapy success. Our study revealed that IL-4 antagonized the stimulatory effect of TNF-α on DC’s migratory and immuno-stimulatory functions in vitro, an effect that might impair the potential efficacy of DCs used as vaccines for cancer immunotherapy in vivo.

Since the first cocktail of cytokines reported by Jonuleit in 1997 [10], numerous methods of inducing DC maturation have been developed [8, 23]. To date, CD40L, TNF-α and a cocktail of cytokines have been applied in clinical trials [24]. TNF-α, one of the important pro-inflammatory cytokines usually present in the maturation cocktail has an effect on phenotypic and functional changes in DC mediated via TNF-RI [9]. The binding of TNF-α to TNF-RI initiates complex signaling events, including protein tyrosine kinase-dependent cascades leading to the activation of NF-κB and AP-1 that regulate the expression of numerous immune/inflammatory response genes including IL-12 p70 and CCR7 [25–28].

Induction of an effective immune response depends on the proper functional maturation of DCs [5, 6]. In cancer, DCs inducing a Th1 response consequently favor an efficient cytotoxic immune response. In our study, the reduced capacity of IL-4^+^-DC to stimulate the proliferation and activation of allogeneic CD4^+^ T cells may be due in part to the low expression of co-stimulatory and MHC II molecules on the surface of these DCs and to a reduced secretion of IL-2. IL4^+^-DC activated allogeneic T cells in an incomplete manner with lower percentage of CD69^+^ and/or CD25^+^ T cells, which could explain the reduced proliferating response. Moreover, IL-4 strongly impaired the expression of CD25 (IL-2-Rα subunit) induced by TNF-α on the surface of DCs. The expression of this molecule was described as a characteristic of fully mature DCs [29–31], and it is considered to be an additional marker for DC quality control [31, 32]. Moreover, the link between the amount of IL-2, INF-γ and T cell proliferation has been demonstrated in human DCs [32]. Finally, IL-4 seems able to antagonize TNF-α-DC maturation by down-regulation of all maturation markers, with a particularly dramatic effect being shown on the CD25 marker.

The secretion of IL-12p70 by DC critically regulates the balance between Th1 and Th2 responses and potently induces IFN-γ-secreting Th1 cells [33]. Our results revealed that TNF-α-matured DCs can secrete IL-12p70 in response to sCD40L stimulation (a setting mimicking a T-cell encounter), which demonstrates that the DC were not exhausted with respect to cytokine production, as was suggested for LPS-matured DCs [34]. Interleukin-4 treatment strongly impaired IL-12p70 secretion and enhanced IL-10 secretion by DC, an effect consistent with the reduced percentage of IFN-γ-positive CD4^+^ T cells and the lower production of IFN-γ on MLR assay. Since the Th2 cytokine IL-5 was weakly detected after co-culture of CD4^+^ T cells with either IL-4^+^- or IL-4^−^-DC, these results suggest that IL-4 reduces the capacity of TNF-α-matured DCs to induce Th1 polarization and thus may decrease the efficiency of the immune response. Our findings are in contrast to some publications reporting a stimulatory effect of IL-4 on the ability of DCs to produce IL-12p70 [16] but in accordance with others [35, 36]. This discrepancy in the regulation of IL-12 secretion by IL-4 reported by many studies could be explained by differences in the origin of the DCs (bone marrow vs. monocytes and murine vs. human) but is more likely to be due to the different maturing agents used. The effect of IL-4 is strong enough to antagonize the allo-stimulation that represents a very strong Th1 condition [37].

The induction of CCR7 expression on the surface of DCs governs their migration from peripheral tissues to lymph nodes [38–40]. DCs respond to the ligands CCL21 and CCL19, which direct them to the peripheral lymphatic vessels and toward the T-cell zones of the draining lymph nodes. In vitro transwell migration assays revealed that IL-4 reduced the migratory capability of TNF-α-matured DCs in response to the two ligands. This effect seems to be related to a strong reduction in CCR7 expression at both the protein and mRNA levels. Our findings are the first to demonstrate that IL-4 antagonizes the stimulatory effect of TNF-α on the expression of CCR7, consequently decreasing DC migratory potential. In previous studies on DC matured with TNF-α, CCR7 cell-surface expression was poorly detected [41, 42], as compared to its detection level in studies involving other maturing agents such as LPS, the maturation cocktail (TNF-α, IL-1β, IL-6 and PGE2), or sCD40L [43–45]. Recently, various reports have indicated that in addition to its role in chemotaxis, CCR7 controls the cyto-architecture, rate of endocytosis, survival, migratory speed and maturation of DCs [39, 46]. By inhibiting CCR7 expression, Interleukin-4 may thus modulate all these different functions in DCs.

Another interesting observation is that the inhibitory effect of IL-4 on CCR7 expression seems to be associated with the use of TNF-α as a maturing agent because IL-4 had little or no effect on LPS or CD40L -matured DCs, respectively. This suggests that IL-4 may interfere more specifically with the TNF-α signaling pathway [13]. Moreover, in TNF-α-matured DC, IL-4 treatment decreased the population of CD83^+^/CCR7^+^ cells and enhanced that of CD83^+^/CCR7^−^ cells, which suggests that IL-4 dissociates the regulation of CCR7 and some phenotypic markers. This hypothesis was confirmed by cells in which IL-4 had little effect on the expression of co-stimulatory molecules, whilst still showing its effect on CCR7 expression. Our results thus reinforce previous observations suggesting that CCR7 expression may be regulated independently of other maturation-associated molecules [47]. Interestingly, we obtained similar results for the regulation of CD25 expression by IL-4, showing an increased population of CD83^+^/CD25^−^ and CCR7^−^/CD25^−^ cells, which suggests a possible associated regulation of CCR7 and CD25 by IL-4.

The mechanism(s) by which IL-4 interferes with TNF-α-induced CCR7 expression is not known. It might be related to the effect of IL-4 on PGE2 synthesis, which is mediated by the down-regulation of enzymes such as phospholipase A2 (PLA2) or cycloxygenase-2 [48]. Prostaglandin E2 has been shown to increase CCR7 expression in DC stimulated with pro-inflammatory cytokines or sCD40L [44]. In our study, replacing IL-4 with IL-13, which enhances PLA2 expression, at least in macrophages [49], and PGE2 in DCs [19], had the same dose-dependent inhibitory effect as IL-4 on CCR7 cell-surface expression, suggesting that the decrease in endogenous PGE2 synthesis was not involved in this process. Previous works investigating chemokine receptor expression on helper T cells indicated the STAT4 dependence of CCR7 expression in mouse Th1 cells [50]. STAT dependence of the CCR7 expression might also be involved in human DCs, connecting both TNF-α and IL4R transduction signals.

## Conclusion

In this study we demonstrated that TNF-α, which could represent a good maturation model in allogeneic response field, appeared as dramatically counter-productive in the induction of mature DCs for cancer therapy. One could speculate that removing only IL-4 from the maturation step will lead to more efficient DCs as they will express a higher mature phenotype, a better T cell stimulating properties and expressing CCR7 allowing their migration.

Finally, our study reinforces the view that the quality of the DC maturation stimulus, which regulates DC migration and cytokine production, may be a decisive feature of the immunogenicity of DCs. Further studies should try to locate the precise regulatory step at which IL-4 seems to interfere with the TNF-α signaling pathway in human DCs.

## Abbreviations

APC: antigen presenting cells
DCs: dendritic cells
GM-CSF: granulocyte macrophage-colony stimulating factor
iDC: immature dendritic cells
IFN-γ: interferon-γ
IL-: interleukin
mAb: monoclonal antibodies
LPS: lipopolysaccharide
MHC: major histocompatibility complex
MLR: mixed lymphocyte reaction
PBMC: peripheral blood mononuclear cells
PG: prostaglandin
PLA2: phospholipase A2
Poly I:C: polyinosinic: polycytidylic acid
sCD40L: soluble CD40 ligand
Th: T helper
TNF-α: tumor necrosis factor-α
